# Occurrence of RNA post-transcriptional modifications in plant viruses and viroids and their correlation with structural and functional features

**DOI:** 10.1016/j.virusres.2022.198958

**Published:** 2022-10-06

**Authors:** Joan Marquez-Molins, Vasti Thamara Juarez-Gonzalez, Gustavo Gomez, Vicente Pallas, German Martinez

**Affiliations:** a Institute for Integrative Systems Biology (I2SysBio), Consejo Superior de Investigaciones Científicas (CSIC) - Universitat de València (UV), Parc Científic, Cat. Agustín Escardino 9, Paterna 46980, Spain; b Instituto de Biología Molecular y Celular de Plantas (IBMCP), Consejo Superior de Investigaciones Científicas (CSIC) - Universitat Politècnica de València, CPI 8E, Av. de los Naranjos s/n, Valencia 46022, Spain; c Department of Plant Biology, Uppsala BioCenter, Swedish University of Agricultural Sciences and Linnean Center for Plant Biology, Uppsala 750 07, Sweden

**Keywords:** RNA, Pathogens, Immune response, Defense, RNA modifications, Epitranscriptomics, Viruses, Viroids, m1A

## Abstract

Post-transcriptional modifications of RNA bases are widespread across all the tree of life and have been linked to RNA maturation, stability, and molecular interactions. RNA modifications have been extensively described in endogenous eukaryotic mRNAs, however, little is known about the presence of RNA modifications in plant viral and subviral RNAs. Here, we used a computational approach to infer RNA modifications in plant-pathogenic viruses and viroids using high-throughput annotation of modified ribonucleotides (HAMR), a software that predicts modified ribonucleotides using high-throughput RNA sequencing data. We analyzed datasets from representative members of different plant viruses and viroids and compared them to plant-endogenous mRNAs. Our approach was able to predict potential RNA chemical modifications (RCMs) in all analyzed pathogens. We found that both DNA and RNA viruses presented a wide range of RCM proportions while viroids had lowest values. Furthermore, we found that for viruses with segmented genomes, some genomic RNAs had a higher proportion of RCM. Interestingly, nuclear-replicating viroids showed most of the predicted modifications located in the pathogenesis region, pointing towards a possible functional role of RCMs in their infectious cycle. Thus, our results strongly suggest that plant viral and subviral RNAs might contain a variety of previously unreported RNA modifications, thus opening a new perspective in the multifaceted process of plant-pathogen interactions.

## Highlights

• RNA modifications were predicted for plant viruses and viroids using a bioinformatic pipeline.
• Plant viruses and viroids present previously unreported RNA modifications.
• RNA modifications of viruses and viroids might have a functional relevance.
• RNA modifications were enriched in the pathogenesis region of nuclear-replicating viroids.

## 1 Introduction

RNA is one of the most versatile molecules of life, being involved in a myriad of biological processes, although many novel and/or unexpected functions are probably yet to be discovered (Sharp, 2009). Similar to proteins and DNA, RNAs can undergo post-transcriptional chemical modifications that alter its structure and, therefore, affect intramolecular interactions, function, and stability, potentially contributing to the regulatory dynamism of RNA (Lewis et al., 2017). To date, around 160 RNA chemical modifications (RCMs) have been described (Boccaletto et al., 2018; Luo et al., 2021). Post-transcriptional modification of RNA bases are widespread across eukaryotes and have been linked to RNA maturation, stability, and interactions with other cellular factors (Nachtergaele and He, 2017). These modifications are highly abundant in transfer RNAs (tRNAs) and ribosomal RNAs (rRNAs) and to a lesser extent in messenger RNAs (mRNAs), long-noncoding RNAs (lncRNAs) and microRNAs (miRNAs) (Shen et al., 2019). Despite the fact that some of these RCMs, such as 5-methylcytosine (m5C) and N6-methyladenosine (m6A), were first detected in the 1970s (Grosjean, 2005; Holley et al., 1965; Perry and Kelley, 1974) the identification of their role has not been possible until the last decade (Marbaniang and Vogel, 2016; Yang et al., 2018a). In plants, the best characterized RCMs are m6A and m5C (Arribas-Hernández and Brodersen, 2020) which might play a role in developmental processes and are enriched in structural features of mRNAs such as the 5′ region and stop codon (Luo et al., 2014; Parker et al., 2020; Wan et al., 2015). m5C methylation, on the other hand, has been shown to guide systemic transport of messenger RNA over graft junctions (Yang et al., 2019).

Although most analysis have focus on the study of post-transcriptional modifications in endogenous RNAs, some of these modifications have been also detected in non-cellular pathogens (Netzband and Pager, 2020; Potužník and Cahová, 2020). Infectious nucleic acids (viruses and viroids) use the host cellular machinery to replicate and transcribe their genomes (García and Pallás, 2015). These pathogens have evolved a diversity of genomic configurations to adapt to their hosts. For example, viruses have developed diverse genomic characteristics, being either DNA or RNA, segmented or non-segmented, double or single stranded of plus or minus polarity (Pasin et al., 2019). On the other hand, viroids (which are the simplest known pathogens) consist of covalently closed single-stranded RNAs (ssRNAs) in the range of a few hundreds of nucleotides (Flores et al., 2015). Recently, post-transcriptional modifications of viral genomes have been identified and linked to different steps of their life cycle. For example, m6A has been extensively reported in animal viruses, having diverse roles, from pro-viral to anti-viral (Williams et al., 2019). In plants, m6A has only been described in two virus of the family *Bromoviridae*, having an anti-viral effect in one of them (Martínez-Pérez et al., 2017) and more recently in two rice viruses from the *Phenuiviridae* and *Spinareoviridae* families (Zhang et al., 2021). Another well studied RCM is m5C, which is also present to high levels in animal viruses compared to endogenous RNAs (Tsai and Cullen, 2020), although it was absent in two representative members of viroids (Di Serio et al., 2019).

The role of RCMs and their enriched presence at certain RNAs has been extensively explored in the last years due to the coupling of RCM-recognizing antibodies with next-generation sequencing (Schwartz and Motorin, 2017). Although these techniques are not able to offer nucleotide resolution, they have contributed to understand the transcriptome-wide distribution of RCMs in different RNA species, such as “housekeeping RNAs” (rRNAs, snoRNAs, snRNAs and tRNAs) as well as mRNAs (McIntyre et al., 2020). Additionally, the use of RNA high-throughput sequencing has highlighted discrepancies between the sequences of transcribed RNAs and their genomic origin, which potentially originate as a result of the interference of post-transcriptional modifications or RNA editing with the generation of high throughput sequencing libraries (Ebhardt et al., 2009). These differences result from the incorporation of random nucleotides at positions where a modified nucleotide is present during the reverse transcription (RT) step needed to produce high-throughput sequencing libraries. Interestingly, this incorporation errors are a valuable source of information for spotting RCMs at single nucleotide-resolution level (Ebhardt et al., 2009). Based on this fact, a software to identify and characterize those modifications was developed, termed HAMR (acronym of: high-throughput annotation of modified ribonucleotides) (Ryvkin et al., 2013; Vandivier et al., 2019). HAMR uses a machine learning step to predict the identity of the RNA modification based on the specific trinucleotide substitution pattern. This algorithm was first validated by the analysis of the substitution pattern in yeast small RNA (sRNA) datasets mapped against known modifications in tRNAs (Ryvkin et al., 2013). Although HAMR is not able to detect modifications that do not alter the base-pairing edge (like m6A), it is capable of identifying 3-methyl cytosine (m3C), 1-methyl guanosine (m1G), and 1-methyl adenosine (m1A) minimizing the number of false positives. In addition, RNA modifications such as pseudouridine (Ψ), *N*^2^-methylguanosine/ *N*^2^,*N*^2^-dimethylguanosine (m2G/m2,2 G), *N*^6^-isopentenyladenosine/ *N*^6^-threonylcarbamoyladenosine (i6A/t6A) and dihydrouridine (D), might be also predicted, but with a minor confidence degree (Ryvkin et al., 2013; Vandivier et al., 2015). HAMR has proven to be useful for the characterization of the Arabidopsis epitranscriptome, where it could identify an enrichment of RNA modifications in alternatively spliced introns and degrading mRNAs and long non-coding RNAs (lncRNAs) (Shen et al., 2019; Vandivier et al., 2015; Vandivier and Gregory, 2018). Nevertheless, whether these modifications are present in plant infectious RNAs was unknown.

Here, we used HAMR to identify and characterize RCMs in plant pathogenic viruses and viroids. We analyzed publicly available sRNA high-throughput sequencing data from representative families of plant viruses and viroids and compared them to endogenous mRNAs. Our analysis was able to detect potential RCMs in all pathogens and identify their correlation with structural features. We further confirmed the presence of some of these modifications in genomic RNAs through RNA immunoprecipitation assay (RIP) from infected tissue followed by PCR-based detection methods. Our study provides a global overview of HAMR-predicted modifications in plant pathogenic viruses and viroids and opens the door to future analysis of their role.

## 2 Material and methods

### 2.1 High-throughput sequence processing and HAMR analysis

High-throughput sequencing sRNA libraries were retrieved from public datasets (Supplementary Table 1). The resulting sequences were trimmed using Trim Galore (Krueger et al., 2021), filtered on length and quality, and concatenated to produce a single sRNA library that contained multiple sRNA libraries using Galaxy (Afgan et al., 2018). These concatenated fastq files were matched to the corresponding virus or viroid genomes using RNA STAR (Dobin et al., 2013) with the following parameters: outFilterType 1, outFilterMultimapScoreRange 1, outFilterMultimapNmax 10,000, outFilterMismatchNmax 10, outFilterMismatchNoverLmax 0.2, outFilterMismatchNoverReadLmax 1, outFilterScoreMin 0, outFilterScoreMinOverLread 0.66, outFilterMatchNmin 0.

The resulting bam file was used as input in HAMR with the following parameters: with min_read_qual 30, min_read_cov 10, seq_error_rate 0.005, hypothesis H4, max_p 0.05, max_fdr 0.05, refpercent 0.05. Viruses and viroids indicated with * in Fig. 2 used low astringent prediction values in HAMR which were the following: min_read_qual 30, min_read_cov 5, seq_error_rate 0.001, hypothesis H4, max_p 0.05, max_fdr 0.05, refpercent 0.05. For visualization of sRNA mapping (Supplemental Fig. 3), sRNA datasets were aligned to the respective pathogen genomes using bowtie (Langmead et al., 2009) allowing 2 mismatches (v2). The genomes of viruses and viroids used in this study is indicated in Supplementary Table 3).

### 2.2 Plant materials and pathogen infections

Hop stunt viroid (HSVd) infected tissues were obtained from *Cucumis sativus* cv. marketer plants agro-inoculated with a dimeric clone of HSVd (genebank accession Y09352.1) as previously described (Marquez-Molins et al., 2019). Samples of systemic leaf tissue were collected 24 days after viroid inoculation. Cucumber mosaic virus (CMV)-infected tissues were obtained from *Arabidopsis thaliana* wild type (ecotype Columbia-0) plants rub-inoculated at the 4 leaves stage (Boyes et al., 2001) with sap material from *Nicotiana benthamiana* infected with CMV (Fny strain). Inoculation was performed using carborundum on the entire surface of the leaves and each leaf was rubbed with approximately 5 μl of sap material diluted in a buffer containing 50 mM TRIS-HCl (pH 8.6) and 50 mM Na_2_HPO. Samples of rosette leaves used for immunoprecipitation analysis were collected at 30 days after viral infection. Initial infection in *N. benthamiana* was performed using transcripts from cDNA clones of the three CMV-Fny genomic RNAs (Rizzo and Palukaitis, 1990). Transcripts were generated using the MAXIscrip T7 Transcription Kit (ThermoFisher). Use of plant materials in this work complies with international, national and/or institutional guidelines.

### 2.3 m1A RNA-immunoprecipitation (RIP) of genomic RNAs

Immunoprecipitation of m1A-enriched viroid RNA was performed as previously described (Dominissini et al., 2013). Total RNA (200 µg) was extracted using TRIzol (Invitrogen) according to the manufacturer's instructions. Those 200 µg of total RNA were incubated in a final volume of 500 µl with 1× IP buffer (50 mM Tris⋅HCl, 150 mM NaCl, 0.5% (vol/vol) Nonidet P-40), 200 U RNasin and 12.5 µg of specific m1A antibody (Diagenode) in rotation for 2 h (h) at 4 °C. Then, 200 μl of protein A dynabeads in 1× IP buffer were added and incubated in rotation for 2 h at 4 °C. After this, beads were washed four times with 1× IP buffer supplemented with Ribolock and eluted. As RIP negative control, the same reaction was incubated with IgG from rabbit serum (Sigma Aldrich-Merck). Immunoprecipitation of m1A-enriched viral RNA was performed using modified protocols from Meyer et al. (2012), Ryvkin et al. (2013) and McCue et al. (2012). In brief, total RNA (100 µg per bioreplicate) was extracted from CMV-infected plants using TRIzol (Invitrogen) according to the manufacturer's instructions. Goat anti-rabbit magnetic beads (New England Biolabs) were washed with IP buffer 1 (1 M NaCl, 10 mM NaH2PO4, 0.05% Triton-X) and pre-incubated with 10 μg of m1A antibody (Diagenode). After pre-incubation, the beads were washed with IP buffer 1 and dissolved in IP buffer 2 (140 mM NaCl, 10 mM NaH2PO4, 0.05% Triton-X) containing 2 µl of RNase inhibitor (Invitrogen). Total RNA (50 µg) were denatured at 75 °C for 5 mins, put on ice for 2 mins and subsequently mixed with IP buffer 2 and incubated with the m1A pre-incubated magnetic beads for 2 h at 4 °C with rotation. After this incubation, beads were magnetically separated, washed with IP buffer 2 and treated with ROTI phenol:chloroform:isoamyl alcohol (ROTH) to extract the bound RNA. As RIP negative control, the same reaction was incubated with IgG from rabbit serum (Sigma Aldrich-Merck). Extracted RNA was used to synthesize cDNA using the RevertAid First Strand cDNA Synthesis Kit (Thermo Scientific).

### 2.4 RT-PCR and RT-qPCR

For RT-PCR analysis, RNA was extracted from antibody-bound beads using 1 ml of TRIzol (Invitrogen) directly on the beads. After pipetting up and down thoroughly, the 1 ml was transferred to a new tube and 200 μl of chloroform were added, mixed using intense vortex for 15 s (*sec*) and incubated at room temperature for 10 min (min). After 15 min of centrifugation at 4 ºC the supernatant was recovered and precipitated with isopropanol, washed with ethanol 75%, resuspended in nuclease free water. An additional precipitation with 10% of AcNa 3 M and 2.5 vol of absolute ethanol was performed for improving the quality. Total RNA was treated with DNAse I, RNAse-free (ThermoFisher) and reverse transcribed using the RevertAid RT Reverse Transcription Kit (ThermoFisher). In brief, reactions were performed at 42 ºC for 1 h with a final step at 70 ºC for 5 min using random hexamers or oligo(dT)18 for the amplification of HSVd or CMV, respectively. PCR was performed with DreamTaq (ThermoFisher) with the primers shown in Supplementary Table 2. PCR conditions steps were 95 ºC for 1 min for initial denaturation followed by a variable number of cycles (indicated in the corresponding figure) that included 95 ºC for 30 s, 55 ºC for 30 s and 72 ºC for 30 s. Reactions were finished at 72 ºC for 10 min. For RT-qPCR a similar strategy was followed although, 10% of the original sample previous to the incubation with antibodies was kept as input. Input samples were used to normalize the expression values of CMV and tRNA Met-CAT using the value of expression of UBIQUITIN10 (AT4G05320) as a housekeeping gene. Immunoprecipitations with m1A and IgG for RT-qPCR were performed in triplicates. Primers used in the qRT-PCR reactions are shown in Supplemental Table 2.

## 3 Results

### 3.1 Analysis of RNA modifications in viruses and viroids using HAMR predictions from sRNA datasets

The use of sRNA high-throughput sequencing data was previously used to predict the presence of RCMs in Arabidopsis mRNAs (Vandivier et al., 2015). We used this experimental approach to infer the presence of putative modified nucleotides in virus and viroid genomes. We retrieved and processed diverse sRNA high-throughput sequencing datasets obtained from different plants infected with viruses and viroids (Supplementary Table 1). In general, viruses and viroids are targeted by the RNA silencing machinery which produces massive amounts of sRNAs derived from their genomic RNAs and/or from genome-derived transcripts (for DNA virus) (Dunoyer and Voinnet, 2005; Gómez et al., 2009). Therefore, sRNA sequencing data constitutes a highly representative sample of the viral and viroid genomes, being a valuable sequence source for HAMR analysis.

**Fig. 1:**
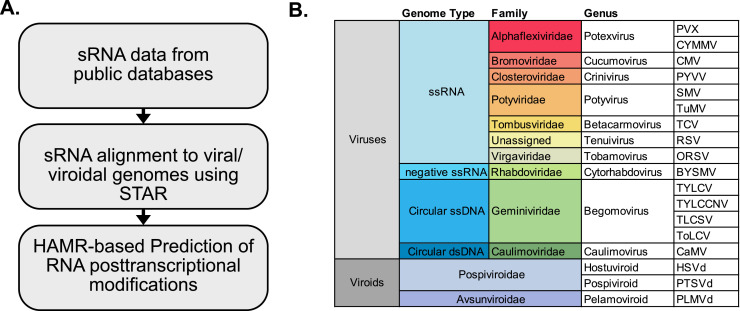
**Computational prediction of nucleotide modifications in virus and viroids. A** Workflow of HAMR analysis for high-throughput annotation of modified residues. **B** Viruses and viroids analyzed.

To use sRNA sequencing data for the prediction of modified nucleotides, we designed a bioinformatic pipeline (Fig. 1A). In particular, we analyzed data from fifteen viruses and three viroids (Fig. 1B). Our analysis included: ssRNA viruses of plus polarity containing representative members of the families *Alphaflexiviridae, Bromoviridae, Closteroviridae, Potyviridae, Tombusvirudae, Virgaviridae* in addition to rice stripe virus (RSV) which is unassigned (Cao et al., 2014; Wu et al., 2016; Yang et al., 2018b); viruses with negative ssRNA genome like barley yellow striate mosaic virus (BYSMV) from the *Rhabdoviridae* family (Yan et al., 2015); DNA viruses which included members from the *Geminiviridae* (ssDNA) (Prakash et al., 2020) and *Caulimoviridae* (dsDNA) families (Leonetti et al., 2021); and the two families of viroids, which were represented by hop stunt viroid (HSVd) and potato spindle tuber viroid (PSTVd) pertaining to the *Pospiviroidae* family (Demian et al., 2020; Marquez-Molins et al., 2021; Owens, 2007; Zheng et al., 2017) and peach latent mosaic viroid (PLMVd) as an *Avsunviroidae* member (Bolduc et al., 2010; Flores et al., 2006). Overall, our analysis provided a general overview of RCMs in plant viruses and viroids.

### 3.2 Characterization of HAMR-predicted modifications in viral and viroid genomes

**Fig. 2:**
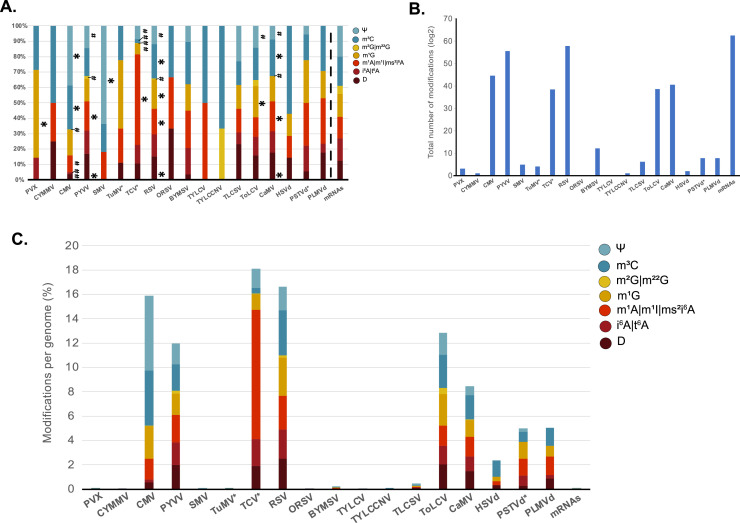
**Characterization of HAMR-predicted modifications in viral and viroid genomes. A.** Identification of RNA modifications in viral and viroid pathogens and *Arabidopsis* mRNAs. **B.** Total number of modifications identified in viral and viroid pathogens and *Arabidopsis* mRNAs. **C.** Total number of modifications identified in viral and viroid pathogens and *Arabidopsis* mRNAs normalized to genome size. *=significantly enriched, #=significantly depleted. Viruses and viroids indicated with * used low astringent prediction values in HAMR as indicated in the Materials and Methods section.

The bioinformatic pipeline detected the presence of potential modifications in almost all viruses and viroids analyzed (Fig. 2A). However, some modifications were not detected in certain viruses and viroids, such as i6A/t6A (CYMMV, TuMV, ORSV, SMV, TYLCCNV, TYLCV and HSVd), Ψ (CYMMV, TuMV, ORSV, PVX, TYLCCNV, TYLCV, HSVd and PLMVd) and m2G|m2,2 G (BYMSV, CYMMV, ORSV, PVX, TuMV, SMV, TLCSV, TYLCV, HSVd, PSTVd and PLMVd) (Fig. 2A). Although the lack of these modifications could have biological meaning, we could not discard that this was due to an insufficient depth of sRNA coverage in the libraries analyzed or because the abundance of these modifications may be low in these pathogens. Globally, the number of modifications found in viruses (between 2 and 2853 modifications in TYLCV and RSV respectively) and viroids (between 7 for HSVd and 18 for PSTVd) was lower than the numbers found for mRNAs (3629 modifications), which were used as plant-endogenous control (Fig. 2B).

In order to compare the occurrence of these modifications, we normalized the results to the genome size of the pathogens (Fig. 2C and Supplementary Fig. 1). In general, sequence analysis of viruses and viroids generated a higher proportion of RCMs (except for ORSV, CYMMV and TYLCV) in comparison with mRNAs of Arabidopsis. This was expected since sRNAs produced from viruses and viroids accumulate at a higher proportion than mRNA-derived sRNAs. Genomic RNAs from RNA viruses (CMV, PYVV, TCV and RSV) and mRNAs produced by DNA virus (CaMV and ToLCV) were the most enriched in nucleotide modifications (approximately 166-fold on average, in comparison with endogenous mRNAs) (Fig. 2C). Viral transcripts from DNA viruses are not only produced from RNA polymerase II, but also from RNA polymerases IV and V (Pol II, IV and V), which might explain why the presence of RCMs was higher than in some canonical Pol II transcripts (Fig. 2C). All viruses contained variable amounts of modifications with TCV, RSV, CMV, ToLCV and PYVV having proportionally higher levels of modifications (approximately 215, 197, 189, 152 and 142-fold, respectively, in comparison with endogenous mRNAs) and CYMMV and ORSV (0.7 and 0.5-fold, respectively, in comparison with endogenous mRNAs) containing the lowest amount of modifications (Fig. 2C). Finally, viroids contained a low number of modifications (7–18), but proportionally higher (between 28 and 60-fold, in comparison with endogenous mRNAs) than some RNA viruses (for example BYSMV, ORSV, TuMV, SMV, CYMMV and PVX). Moreover, PLMVd, the chloroplast-replicating viroid, was slightly enriched in modifications (60-fold more than mRNAs) compared to the two nuclear-replicating viroids analyzed, PSTVd and HSVd (59-fold and 28-fold, respectively, in comparison with endogenous mRNAs). This observation may be linked to the existence of specific RNA editing in the chloroplast (Castandet and Araya, 2011; Takenaka et al., 2013) which has been proposed as a factor contributing to the higher mutation rate of chloroplast-replicating viroids (López-Carrasco et al., 2017). According to these new results, we cannot exclude that the mutation rate of viroids, which has been categorized as the highest reported for any biological entity, might be over-estimated to a certain extent (Gago et al., 2009; López-Carrasco et al., 2017). Some of the modified nucleotides were significantly enriched in viral genomes when compared to endogenous mRNAs (Fig. 2A–C, Fisher exact test with significance level <0.05). CMV, RSV and CaMV were significantly enriched in m3C (Fig. 2A). Additionally, CMV was also enriched in Ψ (Fig. 2A). A common scenario observed in our analysis was the significant depletion of some modifications. For example, several marks were significantly reduced in multiple viruses including m2G/m2,2 G in CMV, PYVV, TCV, RSV and CaMV, and Ψ in PYVV, TCV, RSV, ToLCV and CaMV. Additionally, i6A/t6A, D and m1A/m1I/ms2i6A were significantly reduced in CMV and m1G and m3C were reduced in TCV. In summary, our analysis indicated the presence, enrichment, and depletion of different modifications in viral and viroid genomes.

### 3.3 RNA modifications correlate with structural and functional features of pathogenic viruses and viroids

**Fig. 3:**
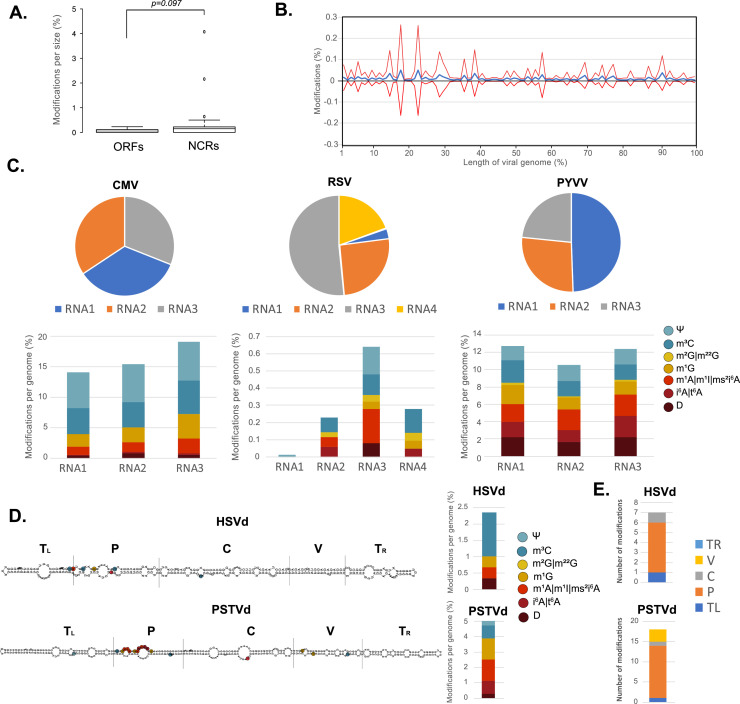
**Genomic localization of the predicted modifications in viroids and viruses with segmented genomes. A.** Presence of modifications expressed as percentage in viral open reading frames (ORFs) or non-coding regions (NCRs). **B.** Distribution of RNA modifications expressed as percentage along the length of viral genomes shown as percentage of the length of the genome (blue line) and extent of the standard deviation of the values (red lines). **C.** Distribution, percentage, and identity of the modifications in the different viral RNAs of CMV (left), RSV (center) and PYVV (right). **D.** Positions with modified nucleotides according to HAMR analysis in the rod-like secondary structure of HSVd and PSTVd. Vertical rows separate the different viroid regions: Terminal left (TL), pathogenesis (P), central conserved region (C), variable (V) and terminal right (TR). **E.** The distribution of the modifications in the different regions shows that are mostly located in the pathogenesis region (71% and 72%, for HSVd and PSTVd, respectively).

In *Arabidopsis*, RNA modifications are associated with alternatively spliced and uncapped mRNAs (Vandivier et al., 2015). Different types of RNA sequencing data show the association of RNA modifications with different structural features of mRNAs (coding sequences and 3′UTR for GMUCT seq and introns for RNA seq). To understand if HAMR-predicted modifications were associated with specific features of viral and viroid genomes, we studied their enrichment at specific features of their genomes (Fig. 3 and Supplementary Table 3). Interestingly, viral open reading frames (ORFs) did not present an enrichment of RNA modifications compared to non-coding regions of their genomes (Fig. 3A). Similarly, we did not observe enrichment of modifications in specific regions of viral genomes (Fig. 3B). The lack of enrichment of modifications in any specific features of the ORFs, suggested that they did not mark specific features of viral mRNAs. This could be observed in detail when the modifications were mapped against the CMV, RSV and PYVV genomes (Supplementary Fig. 2). Nevertheless, multipartite viruses like CMV, RSV and PYVV (Fig. 3C) showed a differential distribution of HAMR-predicted modifications in their genomic RNAs (Supplementary Fig. 2). CMV showed a higher accumulation of modified nucleotides in its RNA3 (19% of the genomic RNA, Fig. 3C), which is required for viral movement and contains its movement and capsid proteins (Roossinck, 2001). RSV showed a higher accumulation of RNA modifications in its RNA3 (0.6% of the respective genomic RNA, Fig. 3C) which codes for its viral silencing suppressor (Cho et al., 2013). This protein is required for the successful infection of the host (Heinlein, 2015). Finally, PYVV showed enrichment of modifications in its RNAs 1 and 3 (13 and 12% of the respective genomic RNAs) which code for its RNA-directed RNA polymerase and capsid protein, respectively (Fig. 3C). Therefore, it is plausible that RNA modifications could be involved in the regulation of the stability and translation of the viral proteins indicated for these three multipartite RNA viruses (CMV, RSV and PYVV).

On the other hand, viroids showed a characteristic enrichment of modifications in their genomes. Viroid genomes contain five well-defined structural domains which are important for different processes (Keese and Symons, 1985). These include two terminal regions (left, TL, and right, TR) and a pathogenic (P), variable (V), and central domains (C). Strikingly, modified nucleotides were significantly enriched in the P domain of both HSVd and PSTVd (Fisher exact test values of 0.0066 and 0.0002 for HSVd and PSTVd respectively, Fig. 3D, E). Indeed, the majority of predicted modifications (71% and 72%, respectively) are located in this domain in both HSVd and PSTVd (Fig. 3D, E). Interestingly, the presence of these modifications is not due to a higher density of sRNA mapping along those regions (Supplementary Fig. 3). On the other hand, we did not identify any enrichment for PLMVd (Supplementary Fig. 4). This fact arises the question of whether there is a link between nuclear viroid pathogenesis and RNA modifications. Potentially, RNA modifications in the P region could impact the interaction with host factors and thus influence the severity of the symptoms. Nonetheless, further experimental evidence is required to fully address this potential relationship. On the whole, the predicted enrichment of RNA modifications in certain structural/functional features of some RNA viruses and viroids points towards a biological role of the modifications in the infectious cycle of these pathogens.

### 3.4 Experimental validation of the presence of m1A in viral and viroid RNAs

**Fig. 4:**
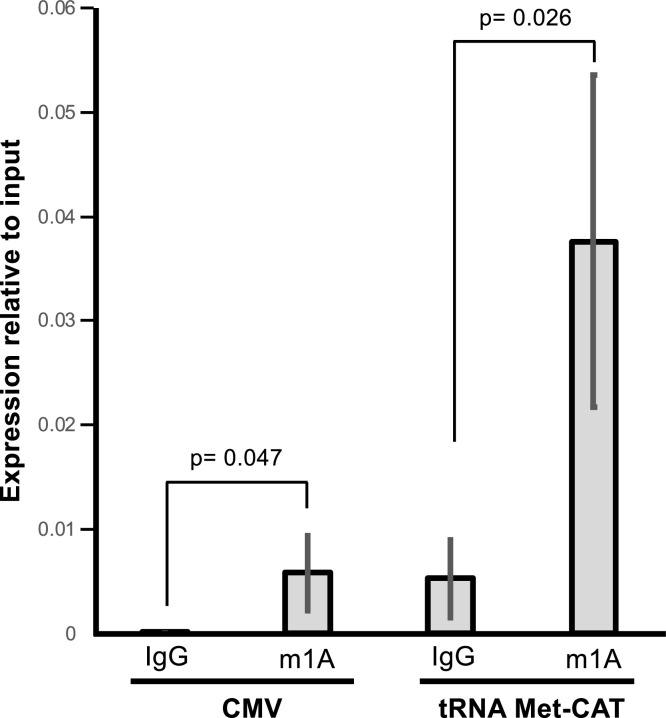
**m1A RNA-immunoprecipitation (RIP) of genomic RNAs of CMV.** Total RNA extracted from plants of *A. thaliana* infected with CMV was used for the RIP taking the 10% as input. The negative control consisted in the same quantity of RNA but incubated with IgG. Immunoprecipitated RNAs with a specific m1A antibody (m1A-IP) or the negative control (IgG-IP) were analyzed by RT-qPCR with specific primers for CMV and a positive control (tRNA Met-CAT). Error bars show the standard deviation values from 3 bioreplicates of RIP.

To provide experimental support to our HAMR-predicted modifications we performed m1A RNA immunoprecipitation (m1A-RIP) followed by PCR-based detection of the RNA genome of samples infected with CMV and HSVd (Supplementary Fig. 5). Our bioinformatic analysis indicated that both pathogenic viruses and viroids showed the presence of m1A, especially CMV (Figs. 2A and 3C) which showed the accumulation of this mark at several positions of its 3 subgenomic RNAs. After m1A-RIP we performed RT-PCR with specific primers for the pathogens in order to identify any potential enrichment of their genomic RNAs. This strategy indicated an enrichment of both CMV and HSVd RNAs in the m1A immunoprecipitated samples compared to the IgG control (Supplementary Fig. 5). To confirm these results, we performed m1A-RIP followed by RT-qPCR in CMV-infected samples (Fig. 4). This technique offers higher level of resolution including quantification of the potential enrichment of specific RNAs. As a positive control, we aimed for the detection of the tRNA methionine anticodon CAT (Met-CAT), which is known to contain the m1A modification (Rashad et al., 2020). Our results showed that both RNAs (CMV and Met-CAT) are significantly enriched following m1A-RIP compared to the use of a non-specific antibody such as IgG (Fig. 4). This analysis confirmed our previous observation of m1A presence in the CMV genome. Furthermore, these results reinforce the validity of HAMR-based prediction of m1A presence in viral and viroid genomic RNAs. In summary, our analysis indicated that HAMR-implementation with sRNA datasets can be a high-throughput method to identify RCMs present in viruses and viroids.

## 4 Discussion

RNA modifications are emerging as important post-transcriptional regulators of cellular life (Luo et al., 2021; Nachtergaele and He, 2017). These modifications have been linked to different steps of viral infection in both animal and plant species, revealing a critical role of this mechanism in the modulation of pathogen-host interactions (Martínez-Pérez et al., 2017; Potužník and Cahová, 2020; Pyle et al., 2019; Williams et al., 2019). Here, we performed an exploratory analysis of the presence of RNA modifications in diverse plant-pathogenic viruses and viroids. We used a HAMR-based approach using public sRNA datasets, which in the past proved to be a reliable source for the prediction of RNA modifications in plant mRNAs (Ryvkin et al., 2013; Vandivier et al., 2015). This approach is especially interesting for pathogenic viruses and viroids, which are active targets of the RNA silencing machinery (Dunoyer and Voinnet, 2005; Gómez et al., 2009). Overall, our analysis showed that representative RNA/DNA viruses and viroids, contained (or produced) RNAs with post-transcriptional modifications. Although in general RNA modifications were not globally overrepresented in pathogenic viruses and viroids, our analysis detected that certain modifications were enriched. This is the case of m1A, the most commonly enriched modification, which was significantly higher in several viruses including PYVV, TCV, RSV and CaMV. Since our approach is based on sRNA sequencing, we cannot exclude that we have overlooked modifications that could not be detected due to a lack of depth in the sRNA high-throughput sequencing datasets used here. On the other hand, since we used a computational prediction, we cannot assume that all the predicted modifications are certainly present. However, most of the modifications should be accurate because HAMR was designed to minimize false positives and has been validated in several organisms (Ryvkin et al., 2013; Vandivier et al., 2015).

RNA modifications are known to mark specific regions of mRNAs and accumulate in uncapped mRNAs in plants (Cui et al., 2017; Vandivier et al., 2015). Our data indicated that HAMR-predicted RNA modifications were also associated with specific features in pathogenic viruses and viroids. For example, in multipartite viruses, we found that some genomic RNAs are enriched in RCMs. This is the case of CMV, RSV and PYVV, whose RNAs coding for proteins involved in viral movement and silencing suppression had a higher number of modifications. These proteins are known to be required for a successful infection process, pointing to a potential role of RNA modifications for the regulation of the translation of those proteins or the regulation of those RNAs. We detected RNA modifications in viroids, including high levels in PLMVd and PSTVd. The high level of RNA modifications present in PLMVd might be linked to the activity of the RNA modification machinery in the chloroplast, the cellular compartment where this viroid family replicates (avsunviroidae). High levels of RNA modifications (in particular RNA methylation) have been detected in chloroplastic RNAs, where they might play an important role for organellar RNA metabolism (Manduzio and Kang, 2021). Intriguingly, we also found an enrichment of RNA modifications in the pathogenic domain of the analyzed viroids of the *Pospiviroidae* family (PSTVd and HSVd). Changes in that domain affect the virulence of the viroid infection (Adkar-Purushothama and Perreault, 2020). Whether the enrichment of modifications in this domain have any significance for viroid virulence is an interesting observation that deserves further investigation in the future. It has been proposed that RNA modifications on viral genomes might prevent the detection of the viral RNAs by the host antiviral RNA immunity mechanism (Gokhale and Horner, 2017). Thus, the presence of these modifications in viral and viroid genomic RNAs could also be involved in the avoidance of the triggering of plant immune response. Together with our prediction we have carried out a confirmation of the enrichment of one of the modifications, m1A, in CMV and HSVd. Our results support the presence of m1A residues in the genome of both analyzed pathogens, which according to RT-PCR analysis, were enriched in the m1A immunoprecipitated fraction compared to control.

## 5 Conclusions

In summary, our work suggests that pathogenic viruses and viroids can contain a variety of RNA modifications at both genomic and/or transcriptomic levels. Furthermore, the relative abundance of these type of epitranscriptomic marks in specific regions and/or structural domains of its genomes, permits to speculate about the possibility that certain modifications might have a functional significance. This considerably extends the knowledge about RCM present in plant pathogens as previously only m6A modification had been identified in some viruses (Martínez-Pérez et al., 2017; Zhang et al., 2021). The modifications identified in our work are targets for future analysis using reverse genetics or direct sequencing of viral and viroid genomes using techniques that allow the detection of these modifications with nucleotide resolution. It is likely that some RCMs affect the interactions with the cellular machinery, promoting the degradation of modified RNAs or favoring interactions which might have a pro-viral result. Therefore, unraveling that multiple, previously unreported, RCMs are present in plant pathogenic viruses and viroids opens a new perspective in the multifaceted process of plant-pathogen interactions.

## CRediT authorship contribution statement

**Joan Marquez-Molins:** Methodology, Writing – original draft, Writing – review & editing. **Vasti Thamara Juarez-Gonzalez:** Methodology, Writing – review & editing. **Gustavo Gomez:** Methodology, Writing – review & editing. **Vicente Pallas:** Methodology, Writing – review & editing. **German Martinez:** Methodology, Writing – original draft, Writing – review & editing.

## Declaration of Competing Interest

The authors declare that they have no known competing financial interests or personal relationships that could have appeared to influence the work reported in this paper.

## Data Availability

Data used in this article is already public. Libraries used are indicated in Supplementary Table 1.

## Appendix Supplementary materials

Image, application 1

Image, application 2

Image, application 3

Image, application 4

Image, application 5

Image, application 6
