# Supplementary figures and images for: Occurrence of RNA post-transcriptional modifications in plant viruses and viroids and their correlation with structural and functional features
Source: Virus Res. 2022 Oct 6;323:198958. doi: 10.1016/j.virusres.2022.198958 (PMC10194119; doi:10.1016/j.virusres.2022.198958)

# Supplementary figure 1

**A.**

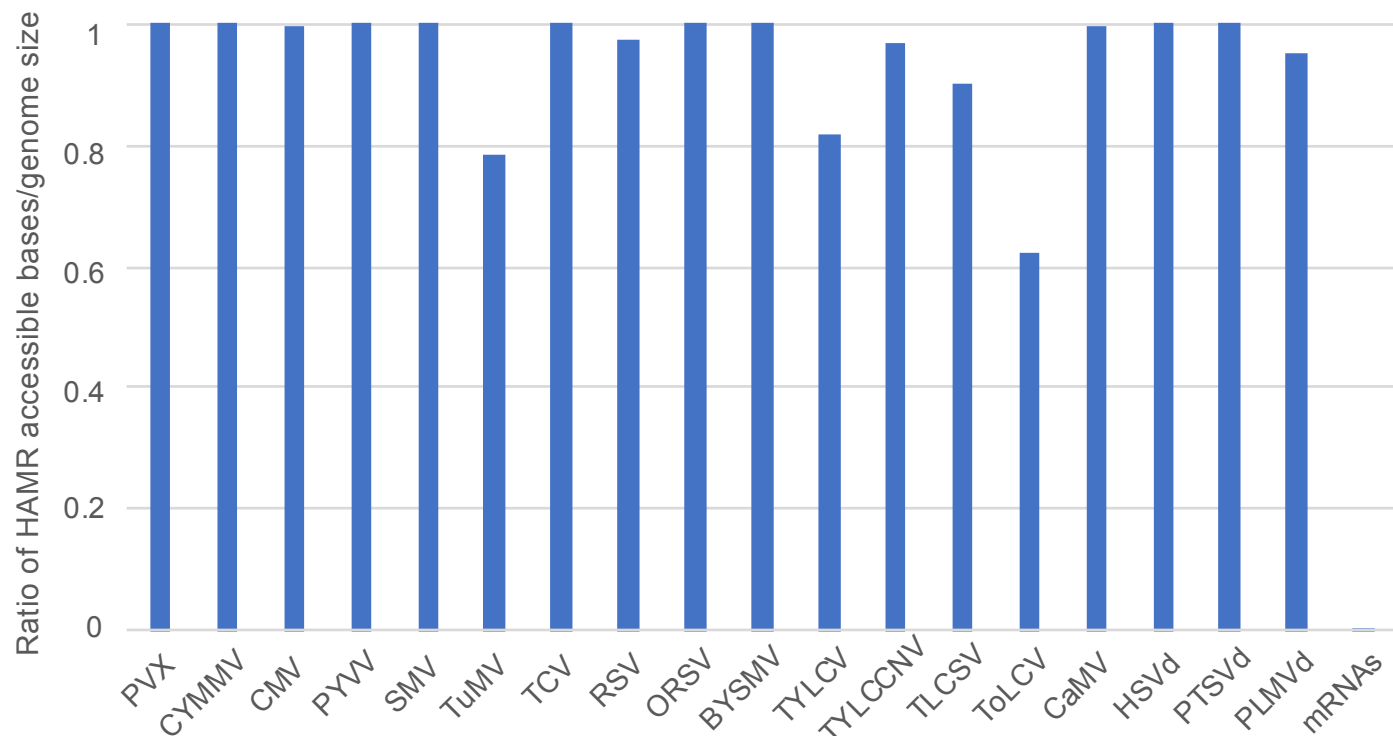

**B.**

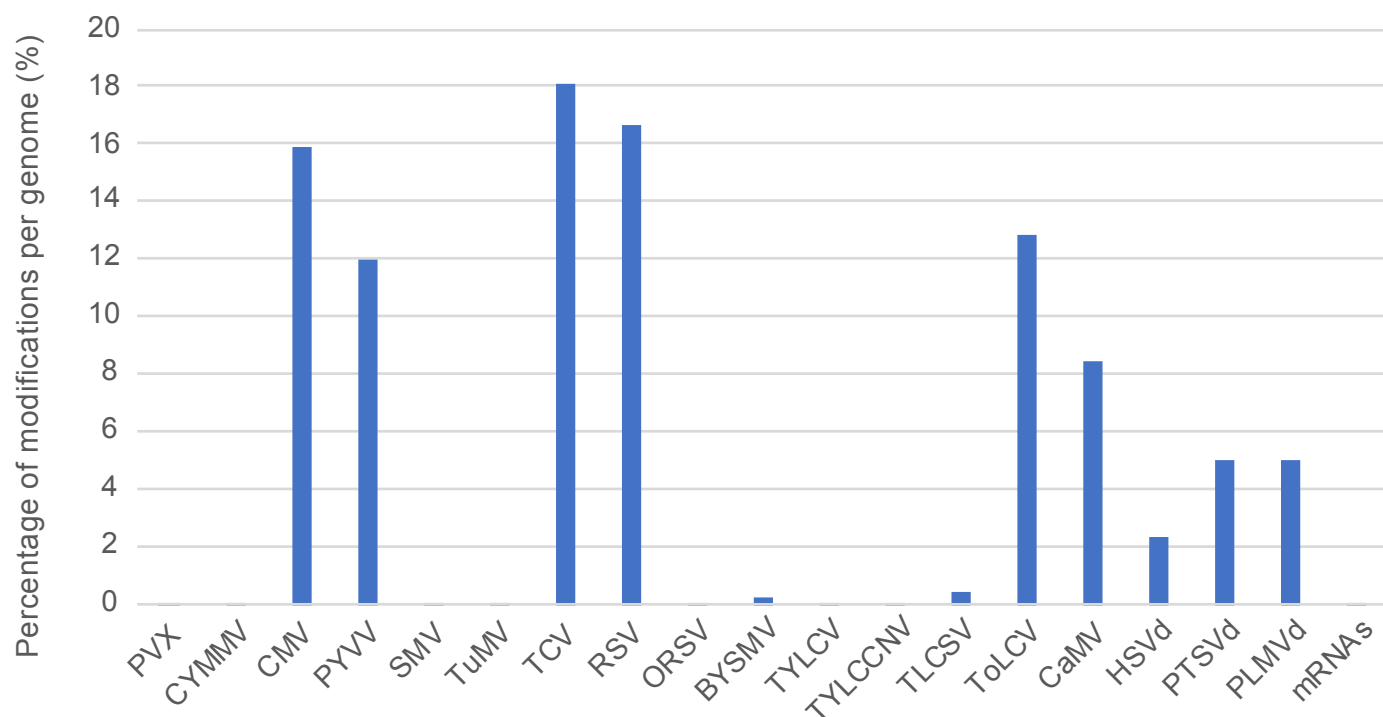

Supplement: Supplementary file 1 [file mmc1.pdf]

Supplementary figure 2

A.

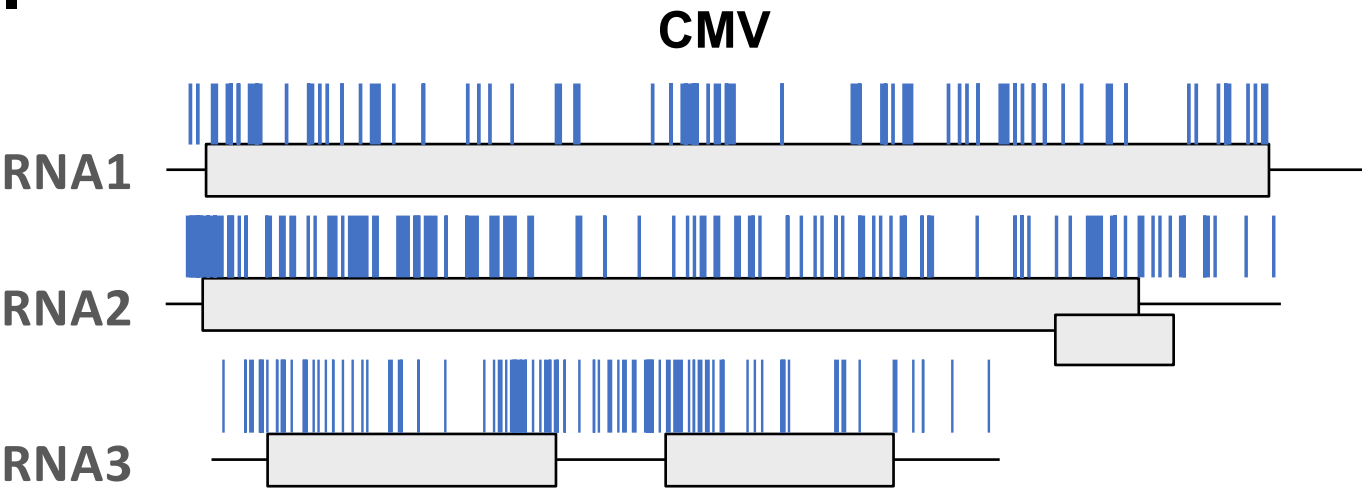

B.

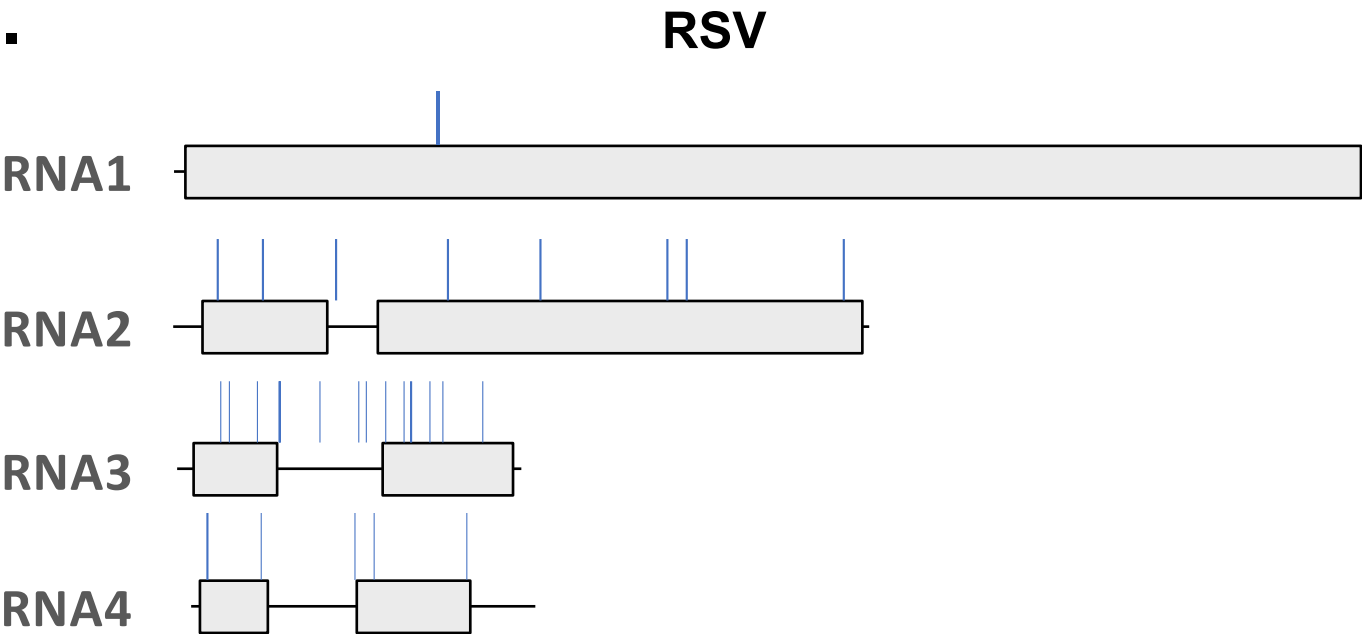

C.

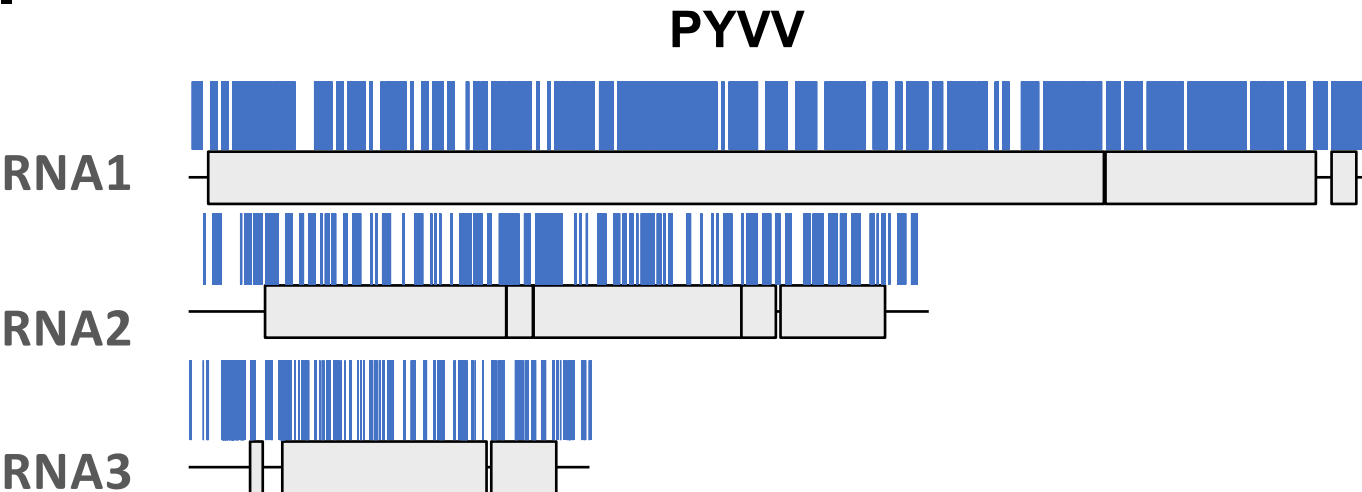

Supplement: Supplementary file 2 [file mmc2.pdf]

# Supplementary Figure 3

**A.**

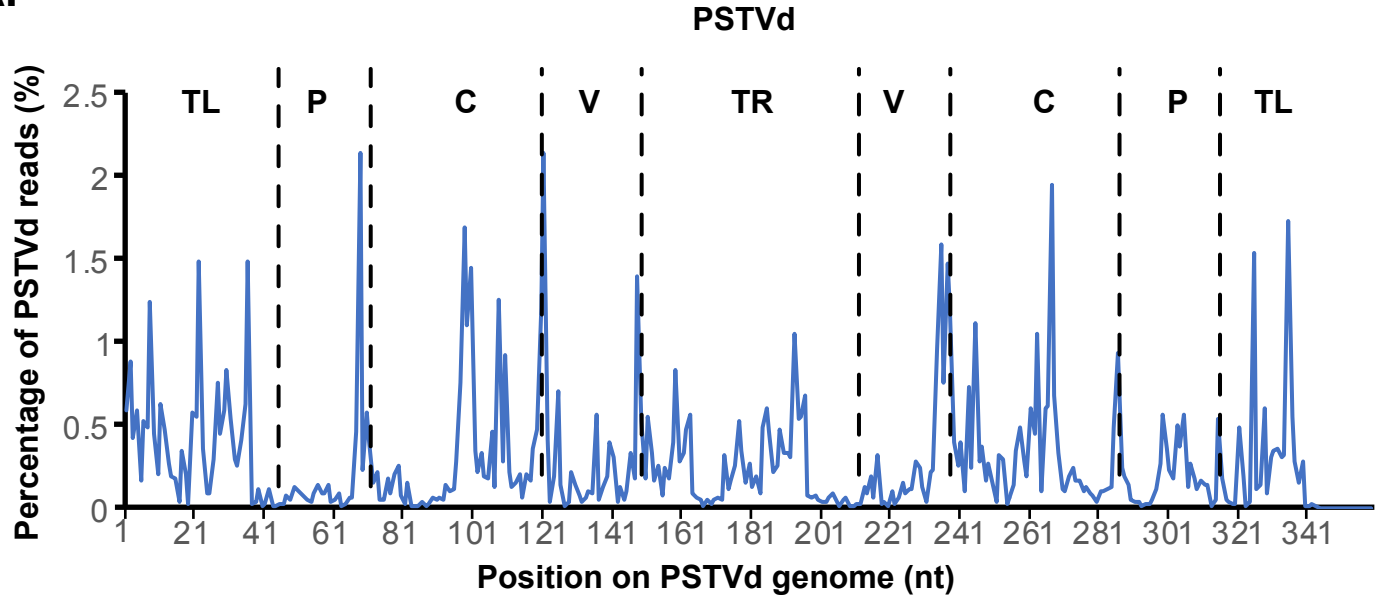

**B.**

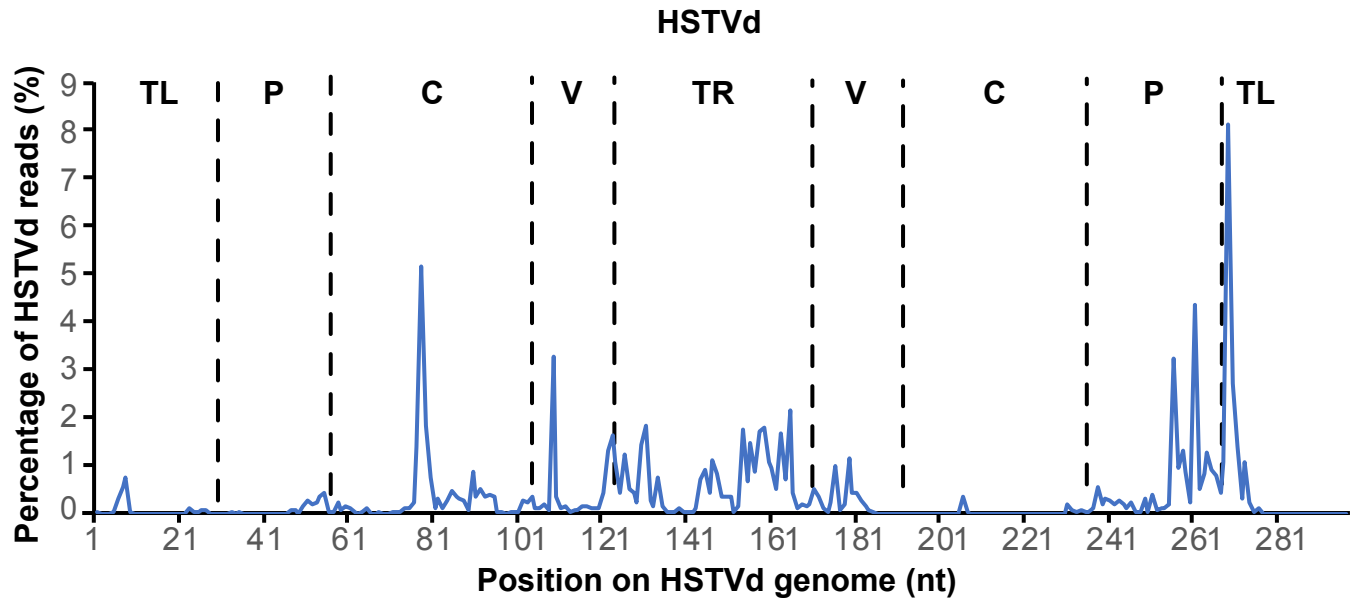

Supplement: Supplementary file 3 [file mmc3.pdf]

Supplementary figure 4

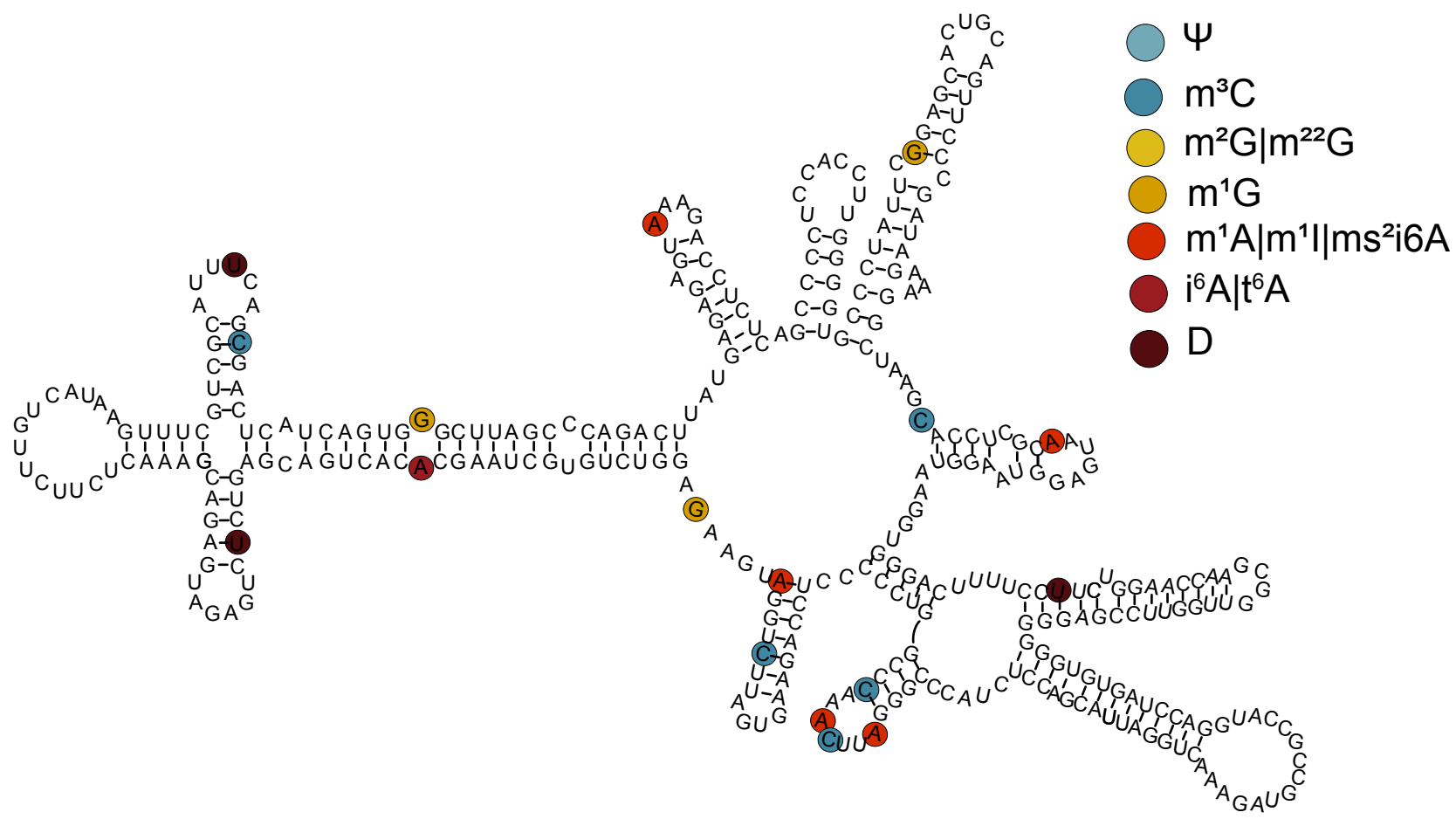

Supplement: Supplementary file 4 [file mmc4.pdf]

# Supplementary Figure 5

**A.**

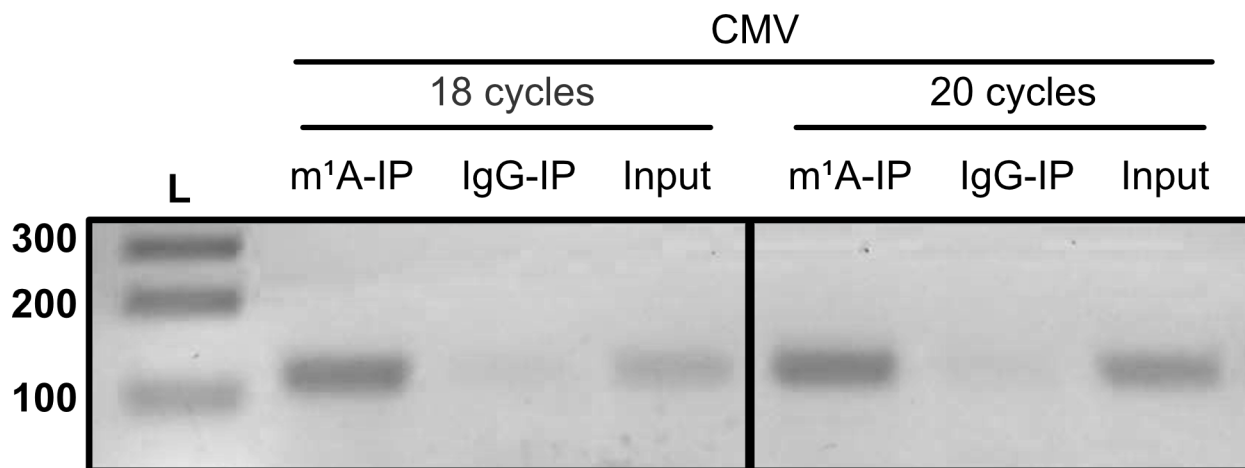

**B**

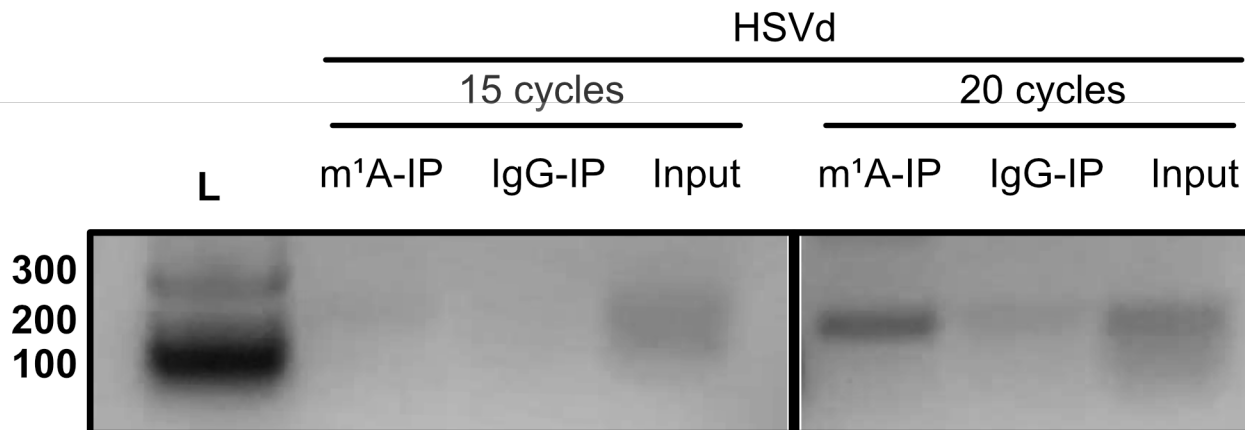

Supplement: Supplementary file 5 [file mmc5.pdf]
